# Mandatory Nicotine Cessation for Elective Orthopedic Hip Procedures Results in Reduction in Postoperative Nicotine Use

**DOI:** 10.7759/cureus.12158

**Published:** 2020-12-18

**Authors:** Brian M Rao, Daniel D Moylan, Kyle R Sochacki, Robert C Kollmorgen, Lakhvir Atwal, Thomas J Ellis

**Affiliations:** 1 Orthopedics, Orthopedic One, Dublin, USA; 2 Orthopedic Surgery, Orthopedic One, Columbus, USA; 3 Orthopedics and Sports Medicine, Houston Methodist Hospital, Houston, USA; 4 Hip Preservation and Sports Medicine, University of California San Francisco, Fresno, USA; 5 Orthopedic Surgery, Orthopedic One, Dublin, USA

**Keywords:** hip preservation, hip replacement, primary arthroplasty, epidemiology, orthopedic, surgery, total hip arthroplasty, smoking, smoking cessation, nicotine

## Abstract

Purpose

To determine the efficacy of mandatory preoperative nicotine cessation on postoperative nicotine use, and to identify independent predictors of nicotine use relapse in subjects undergoing hip preservation surgery or total hip arthroplasty by a single fellowship-trained orthopedic surgeon.

Methods

Consecutive subjects that underwent hip surgery from November 2014 to December 2017 were reviewed. Subjects who self-reported nicotine use, quit prior to surgery, and completed a minimum one-year follow-up were included. Multiple linear regression models were constructed to determine the effect of independent variables on nicotine use relapse following surgery.

Results

Sixty subjects were included in the study (mean follow-up 35.1 months (17-57 months), mean age 44.9 years (20-82 years), and 23 (38.3%) males). Twenty-eight subjects (46.7%) remained nicotine-free at final follow-up. The mean number of cigarettes per day decreased from 13.4 preoperatively to 8.4 postoperatively in the subjects who relapsed (P=0.002). The mean time to return to nicotine postoperatively was 2.4 months. The number of preoperative cigarettes per day was the only independent predictor of tobacco use relapse (P=0.005).

Conclusion

Mandatory preoperative nicotine cessation prior to elective hip surgery demonstrates a 46.7% nicotine-free survivorship at final follow-up with the number of preoperative cigarettes per day found to be the only independent predictor of nicotine use relapse.

Level of evidence

The level of evidence of this research study is Level III since it is a non-experimental study with a cohort of patients.

## Introduction

Tobacco use in the United States remains problematic, despite a long-term decline [1]. It remains the leading cause of preventable death in the U.S. with cigarette smoking responsible for more than 480,000 deaths each year according to the Centers for Disease Control and Prevention (CDC) [2]. Compared to non-smokers, smokers are more likely to develop cancers, cardiovascular disease, pulmonary disease, impaired immune function, and reduced life expectancy [3-5].

Furthermore, smokers are at a higher risk for surgical and postoperative complications [6-11]. Alverdy and Prachand reported that smoking nearly doubles the risk of surgical site infections (SSI) [12]. In an analysis of a large national database, Duchman et al. found that smokers had an increased risk of wound complications and deep infections [13]. The Lower Extremity Assessment Project (LEAP) also found that in open fractures, smokers were twice as likely to develop an infection and 3.7 times more likely to develop osteomyelitis [14]. Evidence suggests that smoking cessation as early as four weeks preoperatively significantly reduces the likelihood of complications, although a timeline for the impact of cessation is lacking [15-19].

The effectiveness of nicotine cessation interventions is not well established. In general, smoking cessation methods have had mixed rates of success. In a meta-analysis, recidivism rates at six months ranged from 69.5% to 88.2% for all cessation methods used and 95% to 97% with self-quitting methods [20]. Ucar et al. reported the recidivism rate of 422 patients using pharmacologic therapies to quit smoking was 65% at 3 months [21].

The preoperative time point has been consistently cited as a “teachable moment” that offers a uniquely effective opportunity for smoking cessation [22-24]. However, many patients quickly return to smoking. Carlson et al. report smoking recidivism rates following spinal surgery of 60% at three months postoperatively, 61% at six months postoperatively, and 68% at one year postoperatively [25]. In smaller studies and systemic analysis, 13-56.3% of patients have achieved long-term cessation at a minimum of one year postoperatively through following various preoperative interventions [23-25].

The effectiveness of nicotine cessation programs in patients undergoing elective hip procedures has not been adequately evaluated. The purpose of the study was to determine the efficacy of mandatory preoperative nicotine cessation on postoperative nicotine use and to identify the independent predictors of nicotine use relapse in subjects undergoing hip preservation surgery or total hip arthroplasty. The authors hypothesized that less than 50% of subjects would remain nicotine-free at final follow up and that there would be no independent predictors of nicotine use relapse.

## Materials and methods

**Figure 1:**
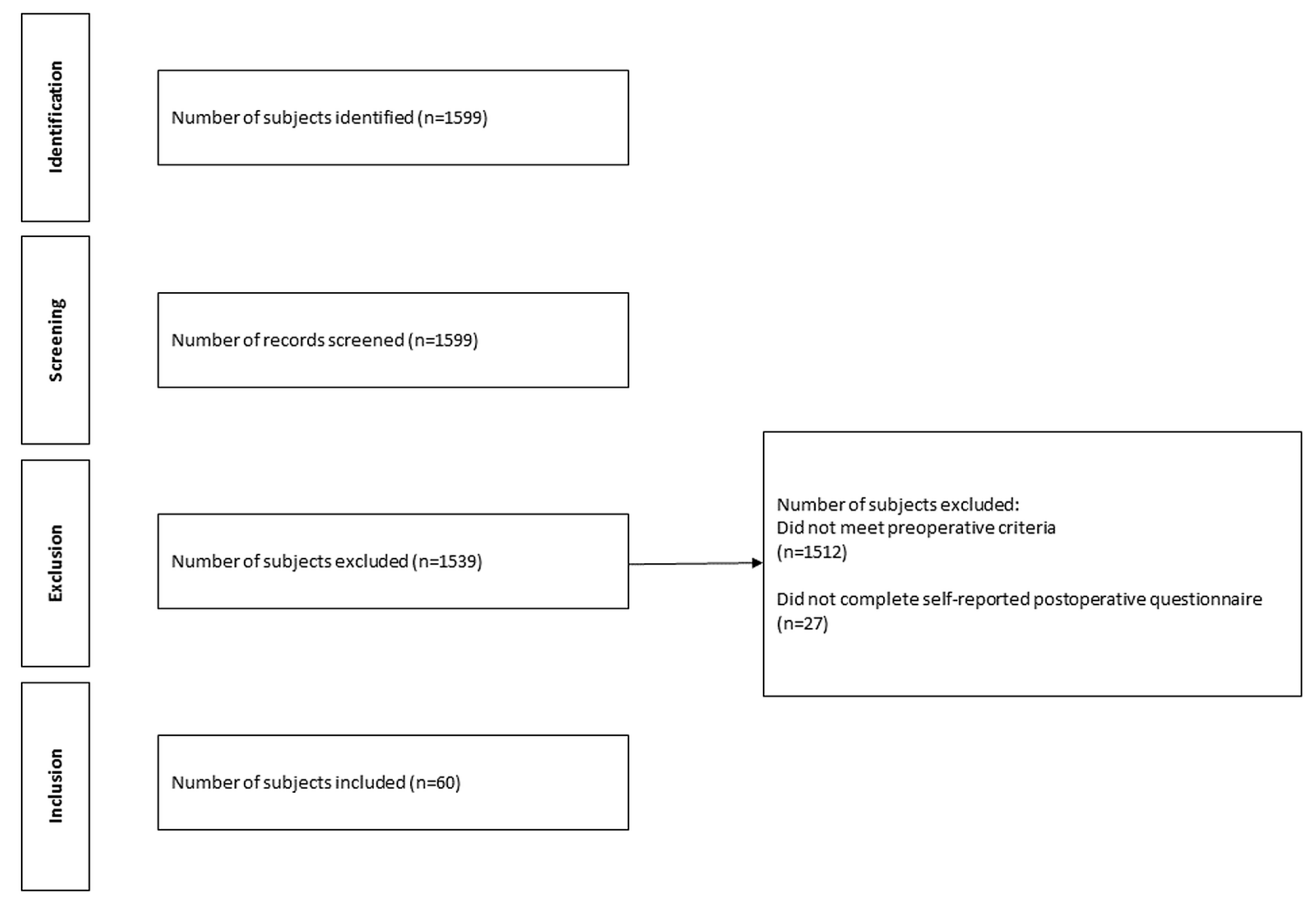
Flow Diagram of Patient Exclusion and Inclusion

Institutional Review Board (IRB) approval was obtained for this retrospective case series. Consecutive subjects who underwent hip preservation surgery or total hip arthroplasty by a single fellowship-trained orthopedic surgeon from September 2014 to December 2017 were reviewed. Subjects aged >18 years old who self-reported nicotine use, quit using nicotine prior to surgery, and completed a minimum one-year follow-up were included in the study. Pediatric subjects and non-native English speakers who could not complete informed consent were excluded (Figure 1).

Self-reported nicotine use was assessed at all patient visits, including their immediate preoperative appointment. Subjects were routinely counseled on the risks associated with nicotine use from smoking, vaping, or chewing tobacco. All subjects undergoing surgery who reported nicotine use were required to quit nicotine use within four weeks of their surgery. This was confirmed by negative serum nicotine/cotinine levels. All subjects were asked to complete a nicotine use survey at their most recent follow-up (Appendices). Subjects who reported nicotine use at any time point after surgery were considered to have relapsed and considered nicotine users.

Subject demographics including age, sex, body mass index (BMI), and surgical procedure was recorded. Nicotine users and nicotine-free subjects were compared. Data analysis was performed using Microsoft Excel for Mac 2018, version 16.16.14 (Microsoft, Redmond, WA, USA). The significance of differences in means of continuous variables between two groups was determined using the Student’s t-test. Categorical variables were compared using Fisher’s exact test. Multiple linear regression models were constructed to determine the effect of independent variables (age, sex, BMI, number of years of prior tobacco use, and the number of preoperative cigarettes per day) on nicotine use relapse following surgery. Categorical variables were coded as dummy variables (i.e., for gender, zero-male, one-female). All P-values were reported, and a significance level of P<0.05 was used.

## Results

Sixty subjects were included in the study (Table 1). The mean postoperative follow-up was 35.1 (12-57) months. The mean age was 44.9 (20-82) years old, with 23 (38.3%) males and 37 (61.7%) females. Procedures performed included 33 total hip arthroplasties (55.0%), 17 (28.3%) hip arthroscopies, 3 (5.0%) iliopsoas tenotomies, 2 (3.3%) periacetabular osteotomies, and 5 (8.4%) other hip preservation-related surgeries. The majority of subjects smoked cigarettes compared to other forms of nicotine preoperatively (86.7%) and postoperatively (78.1%) with most subjects (57.7%) beginning regular nicotine use after the age of 18 (Table 2). Fifty-two (86.7%) subjects quit using nicotine preoperatively unassisted.

**Table 1:** Subject Demographics THA: total hip arthroplasty, PAO: periacetabular osteotomy, BMI: body mass index.

Demographic	All (n=60)	Nicotine Users After Surgery (n=32)	Nicotine-Free After Surgery (n=28)
Mean age (years) (range)	44.9 (20-82)	47.7 (21-80)	41.6 (20-82)
Sex			
Female	37 (61.7%)	20 (62.5%)	17 (60.7%)
Male	23 (38.3%)	12 (37.5%)	11 (39.3%)
Mean BMI (kg/m^2^) (range)	29.6 (18.2-42.1)	30.3 (18.2-42.1)	28.7 (19.6-41.6)
Type of surgery			
THA	33 (55.0%)	17 (53.1%)	16 (57.1%)
Hip arthroscopy	17 (28.3%)	9 (28.1%)	8 (28.7%)
Iliopsoas tendon	3 (5.0%)	3 (9.4%)	-
PAO	2 (3.3%)	-	2 (7.1%)
Other	5 (8.4%)	3 (9.4%)	2 (7.1%)

**Table 2:** Nicotine Use History

Demographic	All (n=60)	Nicotine Users After Surgery (n=32)	Nicotine-Free After Surgery (n=28)
Nicotine products used			
Cigarettes	52 (86.7%)	25 (78.1%)	27 (96.4%)
Chewing tobacco	1 (1.7%)	1 (3.1%)	-
Cigars/pipes	2 (3.3%)	1 (3.1%)	1 (3.6%)
Electronic cigarettes	2 (3.3%)	2 (6.3%)	-
Vaporizers	1 (1.7%)	1 (3.1%)	-
Other	2 (3.3%)	2 (6.3%)	-
Age of regular nicotine use			
18 and under	22 (42.3%)	10 (37.0%)	12 (48.0%)
19 and older	30 (57.7%)	17 (63.0%)	13 (52.0%)
Mean years of prior nicotine use (range)	23.5 (1-50)	23.9 (1-45)	23.5 (3-50)
Mean preoperative cigarettes per day (range)	12.9 (1-40)	13.4 (2-40)	12.3 (1-30)
Mean postoperative cigarettes per day (range)	3.2 (0-40)	8.4 (1-40)	0
Nicotine cessation methods			
“Cold Turkey” no assistance	52 (86.7%)	27 (84.4%)	25 (89.3%)
Nicotine patches	3 (5.0%)	2 (6.3%)	1 (3.6%)
Prescription	2 (3.3%)	-	2 (7.1%)
Tapered down	1 (1.7%)	1 (3.1%)	-
Hypnotism	1 (1.7%)	1 (3.1%)	-
Zero vaporizers	1 (1.7%)	1 (3.1%)	-

Forty (66.7%) subjects either quit nicotine entirely or used less than they did before their surgery. The mean number of cigarettes per day decreased from 13.4 (2-40) preoperatively to 8.4 (1-40) postoperatively in the subjects who relapsed (P=0.002; Table 2). There were no other significant differences (P>0.05) in subject demographics or tobacco use in the nicotine-free group compared to the nicotine users and postoperatively compared to preoperatively.

Twenty-eight subjects (46.7%) were nicotine-free and 32 subjects (53.3%) self-reported as nicotine users at final follow-up. The mean time to return to nicotine use was 2.4 + 2.7 months postoperatively. In multivariate analysis (Table 3), only the number of preoperative cigarettes per day was an independent predictor of nicotine use relapse (P=0.005). No other subject-specific variables had any significant association with nicotine use relapse postoperatively.

**Table 3:** Multivariate Analysis of the Effect of Subject Variables on Nicotine Use Relapse *Statistically significant (P<0.05).

Variable	Regression Coefficient	P-value
Age	−0.045	0.806
Sex	−0.587	0.813
Body mass index	0.372	0.065
No. of years prior nicotine use	0.005	0.979
No. of preoperative cigarettes per day	0.447	0.005*

## Discussion

It was determined that 28 subjects (46.7%) remained nicotine-free at the final follow-up. For the patients who resumed smoking, the mean postoperative number of cigarettes per day significantly decreased compared to preoperatively. Additionally, the number of preoperative cigarettes per day was the only independent predictor of nicotine use relapse. This partially confirmed the authors’ hypotheses.

Postoperative complications associated with nicotine use is a major preventable risk factor. Moller et al. reported numerous complications from smoking tobacco including impaired wound healing, cardiopulmonary complications [9,10], and the need for postoperative intensive care [26]. Smoking cessation four weeks prior to surgery has been shown to positively affect the outcomes of patients and reduce surgical complications [15-19].

Tobacco and nicotine cessation is challenging for an active smoker and often unsuccessful without assistance [26,27]. Prior studies investigating preoperative smoking cessation programs have demonstrated success rates ranging from 32% to 52% at final follow-up [21,23-25,28] with nicotine supplementation found to be the most effective adjunct compared to perioperative education and behavioral therapy. This is similar to the present study in which 46.7% of subjects remained nicotine-free at final follow-up. However, the vast majority of subjects (86.7%) were able to quit “cold turkey” without assistance in the present study.

Additionally, a previous study by Hart et al. demonstrated that greater than 60% of subjects were able to remain tobacco-free at three-year follow-up [28]. The increased tobacco-free survival compared to the present study is likely due to the inclusion of only subjects who were undergoing total knee and total hip arthroplasty while the current study also included less invasive procedures such as hip arthroscopy. Shi and Warner previously demonstrated that more invasive or major surgeries have a greater impact on preventing tobacco use relapse compared to outpatient surgeries such as hip arthroscopy [22].

Mandatory preoperative nicotine cessation also led to reduced nicotine consumption in those subjects who relapsed with the mean number of cigarettes per day decreasing from 13.4 (2-40) preoperatively to 8.4 (1-40) postoperatively. In total, 40 (66.7%) subjects either quitting nicotine entirely or used less than they did before their surgery. This is similar to a prior study by Villebro et al. in which subjects who underwent a preoperative smoking cessation program smoked significantly less cigarettes postoperatively compared to those who did not (10 vs 13) [29]. However, this study only had a long-term tobacco-free survival of 8% at five years in those who were enrolled in a smoking cessation program. It is likely that only offering the subjects a smoking cessation program and not making it mandatory to quit prior to surgery led to the reduced long-term results.

Interestingly, a meta-analysis by Piasecki, found that recidivism rates at six months ranged from 69.5% to 88.2% for all cessation methods [20]. Based on the data from the current study, it would appear that elective orthopedic surgery is an opportune moment to achieve nicotine cessation with recidivism superior to all other methods. Patients are usually receptive to the surgeon educating them on the risks of ongoing tobacco use during the perioperative period. They are also invested in their outcomes and desire to decrease perioperative risks. Therefore, most patients will comply with smoking cessation before surgery. This role of self-preservation is further supported by Shi and Warner. Their study found surgery to be the only independent predictor of successful tobacco cessation compared to other variables [22]. It is possible that combining mandatory preoperative smoking cessation prior to elective orthopedic surgery with a structured counseling or pharmacological program may increase the likelihood that patients will remain nicotine-free. However, this needs further investigation.

The present study also identified the number of preoperative cigarettes per day as the only independent predictor of nicotine use relapse in subjects undergoing elective hip surgery. This was compared to age, sex, BMI, and number of years of prior nicotine use. To the best of our knowledge, no other study in the orthopedic literature has attempted to identify risk factors for relapse. It is not surprising, however, that the more nicotine subjects use, the more likely they are to return to nicotine use postoperatively as quantity may be a surrogate for nicotine addiction [30]. This is compared to the duration of prior use where a subject may only use nicotine occasionally (“social smoker”) despite using nicotine for decades.

There are limitations to this study. The study was retrospective possibly leading to selection bias. All surgeries were performed by a single fellowship-trained orthopedic surgeon with extensive experience in hip preservation surgery and total hip arthroplasty. Thus, the results of this investigation may not be extrapolated to all patient and surgeon populations. Additionally, postoperative nicotine use was based on self-reporting without confirmatory testing. This may have led to underreporting the number of patients who resumed tobacco use following their surgical procedure. 

## Conclusions

In conclusion, mandatory preoperative nicotine cessation prior to elective hip preservation surgery or total hip arthroplasty demonstrates a 46.7% nicotine-free survivorship at final follow-up with the number of preoperative cigarettes per day found to be the only independent predictor of nicotine use relapse. In addition, in the subjects who relapsed, the mean number of cigarettes per day significantly decreased compared to preoperatively. Future work should be done to explore the implementation of more guided cessation methods by healthcare professionals to ease the process for patients as many reported difficulties in quitting.

## Appendices

Patient survey

1.      Email Address

2.      Name

3.      Are you currently using tobacco products? If yes, how long after surgery did you resume?

4.      Are you currently using nicotine vaporizers, electronic cigarettes, or other nicotine products? If yes, how frequently?

5.      Did you use any smoking cessation methods to quit prior to surgery? (Prescription Medication, Nicotine Gum, Nicotine Patches, Vaporizer, Other)

6.      If a smoker, how many years did you smoke prior to surgery? How many cigarettes per day?

7.      Have you tried to quit using tobacco products before? If yes, how many times?

8.      Were there other nicotine users in the household at the time when you quit or currently?

9.      Would you recommend surgeons require patients to quit nicotine use prior to elective surgical procedures?
